# Cell-based analysis reveals that sex-determining gene signals in *Ostrinia* are pivotally changed by male-killing *Wolbachia*

**DOI:** 10.1093/pnasnexus/pgac293

**Published:** 2022-12-13

**Authors:** Benjamin Herran, Takafumi N Sugimoto, Kazuyo Watanabe, Shigeo Imanishi, Tsutomu Tsuchida, Takashi Matsuo, Yukio Ishikawa, Daisuke Kageyama

**Affiliations:** Institute of Agrobiological Sciences, National Agriculture and Food Research Organization, 1-2 Owashi, Tsukuba, Ibaraki 305-0851, Japan; Institute of Agrobiological Sciences, National Agriculture and Food Research Organization, 1-2 Owashi, Tsukuba, Ibaraki 305-0851, Japan; Institute of Agrobiological Sciences, National Agriculture and Food Research Organization, 1-2 Owashi, Tsukuba, Ibaraki 305-0851, Japan; Institute of Agrobiological Sciences, National Agriculture and Food Research Organization, 1-2 Owashi, Tsukuba, Ibaraki 305-0851, Japan; Faculty of Science, Academic Assembly, Toyama University, 3190 Gofuku, Toyama 930-8555, Japan; Graduate School of Agricultural and Life Sciences, University of Tokyo, 1-1-1 Yayoi, Bunkyo-ku, Tokyo 113-8657, Japan; Faculty of Agriculture, Setsunan University, 45-1 Nagaotogecho, Hirakata, Osaka 573-0101, Japan; Institute of Agrobiological Sciences, National Agriculture and Food Research Organization, 1-2 Owashi, Tsukuba, Ibaraki 305-0851, Japan

**Keywords:** cell culture, host sex determination, *Ostrinia scapulalis*, sex-specific gene expression, *Wolbachia*

## Abstract

*Wolbachia*, a maternally transmitted bacterium, shows male-killing, an adaptive phenotype for cytoplasmic elements, in various arthropod species during the early developmental stages. In lepidopteran insects, lethality of males is accounted for by improper dosage compensation in sex-linked genes owing to *Wolbachia*-induced feminization. Herein, we established *Ostrinia scapulalis* cell lines that retained sex specificity per the splicing pattern of the sex-determining gene *doublesex* (*Osdsx*). We found that *Wolbachia* transinfection in male cell lines enhanced the female-specific splice variant of *Osdsx* (*Osdsx^F^*) while suppressing the male-specific variant (*Osdsx^M^*), indicating that *Wolbachia* affects sex-determining gene signals even in vitro. Comparative transcriptome analysis isolated only two genes that behave differently upon *Wolbachia* infection. The two genes were respectively homologous to *Masculinizer* (*BmMasc*) and *zinc finger-2* (*Bmznf-2*), male-specifically expressed sex-determining genes of the silkworm *Bombyx mori* that encode CCCH-type zinc finger motif proteins. By using cultured cells and organismal samples, *OsMasc* and *Osznf-2* were found to be sex-determining genes of *O. scapulalis* that are subjected to sex-specific alternative splicing depending upon the chromosomal sex, developmental stage, and infection status. Overall, our findings expound the cellular autonomy in insect sex determination and the mechanism through which sex is manipulated by intracellular selfish microbes.

Significance Statement In various arthropods, males are killed by maternally inherited *Wolbachia* endosymbionts during early developmental stages. In lepidopteran insects, this is accounted for by improper dosage compensation in males, wherein *Wolbachia* alter the host sex determination from male to female. The present study revealed that *Wolbachia* can even change the sex determination of the cultured cells. The influence of *Wolbachia* on sex determination in cell lines could open the door for future study of *Wolbachia* and for understanding the relationship between *Wolbachia* and its host.

## Introduction

In many eukaryotes, differences in sex can be caused by numerous molecular and physiological pathways during developmental processes (1). Such sophisticated mechanisms can be manipulated or overridden by cytoplasmic elements that are transmitted exclusively from mothers to offspring, resulting in a female-biased sex ratio that is evolutionarily advantageous for the cytoplasm (2, 3). In arthropods, various species of vertically transmitted bacteria residing in the cytoplasm cause bias in the host sex ratio toward females through male-specific lethality during early developmental stages, typically during late embryonic or early larval stages (4). Male-killing bacteria are taxonomically diverse: *Wolbachia* and *Rickettsia* belonging to Alphaproteobacteria (5–8), *Arsenophonus* belonging to Gammaproteobacteria (9), *Spiroplasma* belonging to Mollicutes (10–12), and an uncharacterized bacterium belonging to Flavobacteria (13), all cause male lethality in their respective hosts.

Recent studies have suggested that the mechanisms underlying male lethality are diverse; *Spiroplasma*-induced male lethality in *Drosophila melanogaster* is actuated by male-specific aberrant apoptosis and neural defects in embryos because of DNA damage and segregation defects in the male X chromosome (10, 14–16); *Wolbachia*-induced male-killing in *Drosophila bifasciata* also involves apoptosis, DNA damage and segregation defects in the male X chromosome, but normal neural development is observed (17); *Arsenophonus*-induced male-killing in the parasitic wasp *Nasonia vitripennis* is induced by inhibition of the formation of maternal centrosomes—organelles specifically required for early male embryonic development—resulting in developmental arrest well before the establishment of somatic sexual identity (9).

By contrast, *Wolbachia*-induced male lethality in lepidopteran insects such as *Ostrinina* and *Homona* is associated with the alteration of sex determination from male to female (18–21). In these moths, irrespective of their sex chromosome constitution (i.e., WZ or ZZ), all embryos produced by *Wolbachia*-infected females express the female-specific splicing variant (*dsx^F^*) of *doublesex*, a conserved gene located at the bottom of the sex-determining gene cascade. It has been assumed that *Wolbachia*-infected ZZ individuals (genetic males expressing *dsx^F^*) die because of the global overexpression of Z chromosome-linked genes, that is, improper dosage compensation (20–22). Therefore, at least in *Ostrinia* and *Homona*, and probably more widely in Lepidoptera, alteration of sex determination underpins the phenotypic outcomes (i.e., male-killing). Although these ZZ individuals expressing *dsx^F^* die before or soon after egg hatching, the manipulated *dsx* splicing could be associated with the sexual phenotype; sexually mosaic phenotypes arise in the surviving ZZ offspring produced by females incompletely cured of *Wolbachia* infection (18, 23–25). Notably, male lethality in *Drosophila* does not involve alteration in sex determination (26). Furthermore, Fukui et al. (20) used transcriptome analysis of *Ostrinia furnacalis* embryos to demonstrate that *Wolbachia* reduced the mRNA levels of *Masculinizer* (*OfMasc*), a Z-linked CCCH-type zinc finger motif encoding gene required for both masculinization and dosage compensation in the silkworm *Bombyx mori* (27). Suppression of *OfMasc* by *Wolbachia* was also supported by the fact that injection of in-vitro transcribed *Masc* RNA into *Wolbachia*-infected *O. furnacalis* embryos rescued males (20). In *B. mori*, an autosomal CCCH-type zinc finger motif encoding gene *z2* (*Bmznf-2*) is known to enhance *dsx^M^* and suppress *dsx^F^* when overexpressed in the cell line (28). It was also shown that the knockout of *znf-2* in males enhanced *dsx^F^* and showed weak mating behavior and feminized external genitalia (29). However, nothing is known about the relationship between *znf-2* and *Masc* in *B. mori* and whether a *znf-2* homolog is influenced by *Wolbachia* infection.

Another aspect of *Wolbachia*-induced male lethality in *Ostrinia*, but not in *Homona*, is that the females cured of *Wolbachia* infection using antibiotics produce offspring that only produce males (18, 30). In *O. scapulalis*, all embryos produced by *Wolbachia*-eliminated females were shown to express *dsx^M^* irrespective of their sex chromosome constitution (i.e., either WZ or ZZ) (19). In contrast to infected embryos, WZ individuals with *dsx^M^* die before or just after egg hatching, and the expression of Z-linked genes in these individuals is downregulated, suggesting improper dosage compensation (20, 22). It is likely that the female-determining function linked to the W chromosome is degraded owing to the long-term coexistence with feminizing *Wolbachia*; in other words, the eroded sex-determining system of the infected lineage is complemented by *Wolbachia* (19). In *Homona*, however, elimination of *Wolbachia* reverts the sex ratio to 1:1 (21), suggesting that the *Wolbachia*–*Homona* relationship is not sufficiently long to erode the sex-determining system.

Studies have shown that *Wolbachia* affect the molecular pathway of sex determination at the organismal level, but whether this effect can be reproduced in a cell culture system remains unknown. Compared with in-vivo systems in organisms, cell cultures are genetically homogeneous, which allows to dictate gene expression changes in a detailed manner. Therefore, using cell lines newly established from uninfected males and females of *O. scapulalis*, we tested whether sex determination alteration (i.e., feminization) can be induced in cell culture systems by transinfecting *Wolbachia* derived from *O. scapulalis* (*w*Sca).

## Results and discussion

### Sex identity was retained in newly established cell lines

**Fig. 1.**
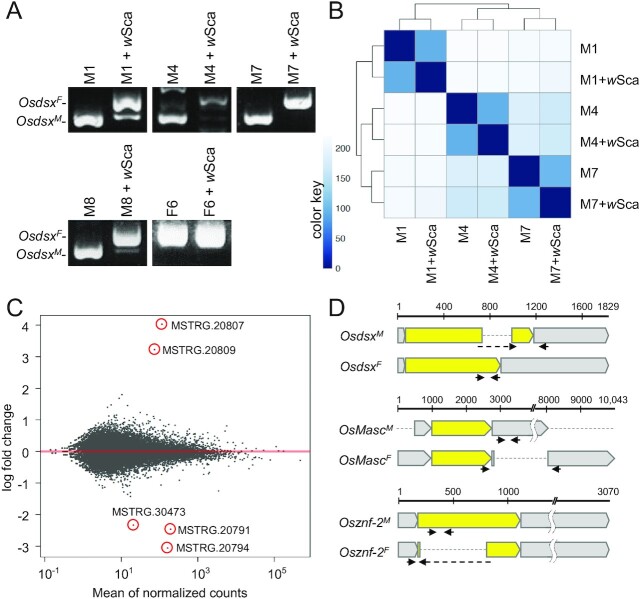
Cell-based analyses of the effects of *Wolbachia* infection on gene expression. (A) Splicing pattern of *Osdsx* in cell lines based on RT-PCR. (B) Heatmap2 analysis showing sample-to-sample distances on the matrix of variance stabilized data for overall gene expression using principal component analysis (PCA). Darker colors indicate similar expression, and color key values indicate arbitrary units. (C) MA-plot showing log fold-changes between uninfected and *w*Sca-infected male cell lines. Each dot indicates a single contig. Red dots surrounded by circles are contigs that showed significant changes upon *w*Sca infection. (D) mRNA structure of *Osdsx^M^, Osdsx^F^, OsMasc^M^, OsMasc^F^, Osznf-2^F^*, and *Osznf-2 ^M^*. Yellow: coding region. Gray: untranslated region. Arrows: position of qPCR primers that specifically amplify sex-specific isoforms of *Osdsx, OsMasc*, and *Osznf-2*.

We established five cell lines of *O. scapulalis*: two from the fat bodies of the last-instar male larvae [NARO-Ossc-M1 (M1) and NARO-Ossc-M8 (M8)], one from the testes of a last-instar male larva [NARO-Ossc-M7 (M7)], one from the testes of a male pupa [NARO-Ossc-M4 (M4)], and one from the fat bodies of a last-instar female larva [NARO-Ossc-F6 (F6)] (Fig. S1). RT-PCR was used to show that the splicing patterns of *Osdsx* were consistent with their original sex (i.e., M1, M4, M7, and M8 expressed *Osdsx^M^* and F6 expressed *Osdsx^F^*) (Fig. 1A). Because *Osdsx* is located at the bottom of the sex-determining gene cascade (31), a suite of sex-determining genes is likely to be expressed canonically in the cell lines, as in the original animal. This was also the case for the *B. mori* cell culture, wherein male-derived and female-derived cells continued to express the male- and female-specific variants of *dsx* (*Bmdsx*), respectively (32).

### Transinfection with *w*Sca feminized the splicing pattern of *Osdsx* in male cell lines

The five cell lines (M1, M4, M7, M8, and F6) were used to transinfect *w*Sca. After confirming by PCR that *w*Sca was stably maintained in each cell line, RT-PCR revealed that all cell lines (M1, M4, M7, M8, and F6) expressed *Osdsx^F^* (Fig. 1A). By contrast, intact cell lines continued to express sex-specific *Osdsx* (*Osdsx^M^* in M1, M4, M7, and M8 and *Osdsx^F^* in F6). These results suggest that *Wolbachia* alters the sex-determining signal cascade from that for male to that for female, even in vitro.

### Global patterns of gene expression were specific to cell lines rather than to *Wolbachia* infection status

RNA-seq analyses were performed using six cell lines: *w*Sca-transinfected M1, M4, and M7, expressing only *Osdsx^F^*, and intact M1, M4, and M7, expressing only *Osdsx^M^*. Sequencing reads from the six cell lines were mapped to *O. furnacalis* genome assembly in the NCBI RefSeq database (GCF_004193835.1). Comprehensive differential gene expression analysis of 56,026 contigs revealed that the global pattern of gene expression was mostly dictated by the origin of the cell lines (M1, M7, or M10) rather than the *Wolbachia* infection status (Fig. 1B). The finding that *Wolbachia* infection does not contribute toward global gene expression profiles is consistent with previously published microarray data, which indicated no differentially expressed genes between *Wolbachia*-infected and uninfected *B. mori* cell lines, NIAS-Bm-aff3 (33).

### Identification of genes whose transcription patterns are altered by *w*Sca transinfection

Among the 56,026 contigs, we found only five contigs (MSTRG.20791, MSTRG.20794, MSTRG.20807, MSTRG.20809, and MSTRG.30473) that consistently showed significant changes in titers in M1, M4, and M7 upon *w*Sca transinfection (Fig. 1C; Fig. S2). MSTRG.20807 and MSTRG.20809 showed upregulation, whereas MSTRG.20791, MSTRG.20794, and MSTRG.30473 showed downregulation upon infection. Analysis of the genome data of *O. furnacalis* and RT-PCR using the primers designed on the contig sequences revealed that four contigs (MSTRG.20791, MSTRG.20794, MSTRG.20807, and MSTRG.20809) were derived from *OsMasc* and one (MSTRG.30473) was derived from *Osznf-2*. Both *OsMasc* and *Osznf-2* were alternatively spliced depending on the *Wolbachia* infection status, that is, *OsMasc^I^* and *Osznf-2^I^* in infected cells and *OsMasc^U^* and *Osznf-2^U^* in uninfected cells (Fig. 1D). Previous studies have assumed that both *Masc* and *znf-2* are male-specific genes that act epistatically on *dsx* (20, 26, 28, 29, 33). However, since the female cell line, F6, expressed *OsMasc^I^* and *Osznf-2^I^*, but not *OsMasc^U^* or *Osznf-2^U^*, we assumed that *OsMasc* and *Osznf-2* are spliced in a sex-specific manner and that *OsMasc^I^*/*OsMasc^U^* corresponds to *OsMasc^F^*/*OsMasc^M^* and *Osznf-2^I^*/*Osznf-2^U^* corresponds to *Osznf-2^F^*/*Osznf-2^M^*. As shown in Fig. 1D, the predicted coding sequences of *Osdsx* and *Osznf-2* were different between sex-specific splice variants, whereas the predicted coding sequences of *OsMasc* splice variants were identical, suggesting that they may function as noncoding RNAs. Long noncoding RNAs, such as *roX* in *Drosophila* and *Xist* in mammals, play important roles in dosage compensation (34). In *B. mori*, RNAi-mediated knockdown of *Masc* in males (ZZ) resulted in the upregulation of many Z chromosome-linked genes, suggesting that *Masc* is necessary for proper dosage compensation (27).

### Chronological changes in the splice patterns of *Osdsx, OsMasc*, and *Osznf-2* in cell lines upon *w*Sca transinfection

**Fig. 2.**
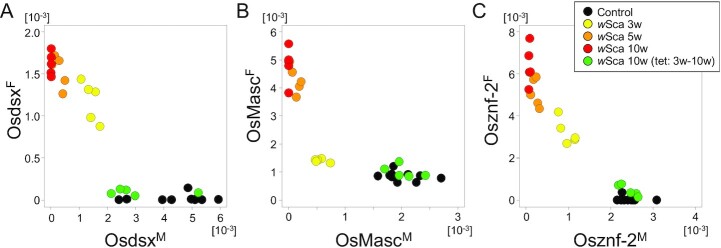
Relative titers of the sex-specific isoforms of *Osdsx* (*A*), *OsMasc* (*B*), and *Osznf-2* (*C*) in the male-derived *O. scapulalis* cell line M4. Black: nontreated. Yellow: 3 weeks after transinfection with *w*Sca. Orange: 5 weeks after transinfection with *w*Sca. Red: 10 weeks after transinfection with *w*Sca. Green: tetracycline treatment, started at the third week after transinfection with *w*Sca and continued for 7 weeks thereafter.

Next, we examined the chronological changes in the sex-specific splice patterns of *Osdsx, OsMasc*, and *Osznf-2* upon *w*Sca transinfection into the M1 and M4 cell lines. A male expression pattern (high expression of *Osdsx^M^, OsMasc^M^*, and *Osznf-2^M^*; marginal expression of *OsMasc^F^*; and no expression of *Osdsx^F^* and *Osznf-2^F^*) was observed before transinfection (Fig. 2). After 3 weeks, *Osdsx^M^, OsMasc^M^*, and *Osznf-2^M^* showed a significant decrease in expression (for both, *P* < 2.2 × 10^−16^ using generalized linear models). At this stage, the expression of *Osdsx^F^* and *Osznf-2^F^* was already induced (*P* = 3.54 × 10^−5^ and *P* = 6.72 × 10^−6^ using generalized linear models, respectively), whereas *OsMasc^F^* expression was slightly but clearly upregulated (*P* = 0.00215 using generalized linear models). Five weeks after infection, a drastic upregulation in the expression of *Osdsx^F^, OsMasc^F^*, and *Osznf-2^F^* and downregulation in the expression of *Osdsx^M^, OsMasc^M^*, and *Osznf-2^M^*were observed. A feminized expression pattern was evident 3 weeks after infection and was almost complete 5 weeks after infection (only marginal increases were observed after 5 weeks) (Fig. 2). To further demonstrate that feminized expression can be cancelled by removing *w*Sca, the cell lines were treated with tetracycline in parallel from the third week of transinfection. Seven weeks after the initiation of tetracycline treatment, the splicing pattern reverted to that of the males (Fig. 2). These results strongly suggest that *Wolbachia* feminizes the splicing patterns of *Osdsx, OsMasc*, and *Osznf-2* in the male cells. The above experiments were also conducted using M1, and the results were considerably similar (Fig. S3). When tetracycline treatment was started 5 weeks after *w*Sca introduction, complete cancellation of feminized expression did not occur even after 12 weeks of tetracycline treatment (Fig. S4). This may indicate the involvement of an autoregulatory loop as a mechanism of sex determination.

### *Osdsx, OsMasc*, and *Osznf-2* are sex-specifically spliced during postembryonic development

**Fig. 3.**
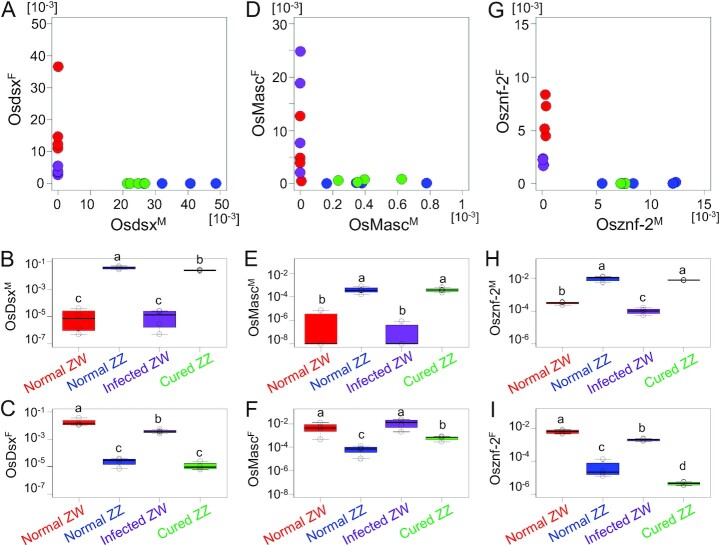
Relative titers of sex-specific isoforms of *Osdsx, OsMasc*, and *Osznf-2* in *O. scapulalis* adults. A, D, and G: titers of sex-specific isoforms of each sample of normal ZW female (red), normal ZZ male (blue), *Wolbachia*-infected ZW female (purple), and cured ZZ male (green). B, C, E, F, H, and I: titers of each isoform compared between normal ZW female (red), normal ZZ male (blue), *Wolbachia*-infected ZW female (purple), and cured ZZ male (green) individuals.

Because we used a simplified model of cell culture, we verified whether it reflected the phenomena occurring in the intact animal from which the cell lines were established. We first examined the expression levels of *Osdsx, OsMasc*, and *Osznf-2* in *O. scapulalis* adults (Fig. 3A, D, and G). The titers of *Osdsx^M^, OsMasc^M^*, and *Osznf-2^M^* in normal males (ZZ) and cured males (ZZ) were consistently higher than those in normal females (WZ) and infected females (WZ) (Fig. 3B, E, and H). Conversely, titers of *Osdsx^F^, OsMasc^F^*, and *Osznf-2^F^* in normal females (WZ) and infected females (WZ) were consistently higher than those in normal males (ZZ) and cured males (ZZ) (Fig. 3C, F, and I). These results in vivo clearly show that *Osdsx^M^, OsMasc^M^*, and *Osznf-2^M^* are male-specific, whereas *Osdsx^F^, OsMasc^F^*, and *Osznf-2^F^* are female-specific. Chronological observations during postembryonic development revealed that the expression patterns of *Osdsx, OsMasc*, and *Osznf-2* were almost identical between uninfected WZ and infected WZ (female), as well as between uninfected ZZ and cured ZZ (male) organisms, although there were some differences in the expression trends of the genes and variants (Fig. S5).

**Fig. 4.**
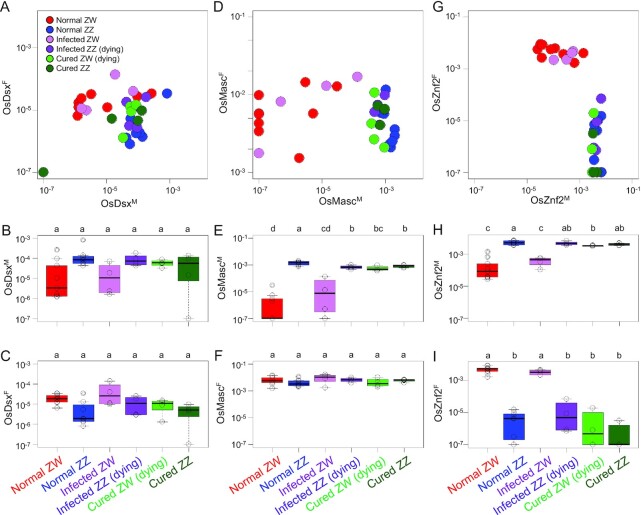
Relative titers of sex-specific isoforms of *Osdsx, OsMasc*, and *Osznf-2* at the first-instar larval stage. A, D, and G: titers of sex-specific isoforms of each sample. B, C, E, F, H, and I: titers of each isoform compared between ZW female (red) and ZZ male (blue) of an uninfected line, ZW (light purple), and ZZ (dark purple) of a *Wolbachia*-infected matriline, and ZW (light green) and ZZ (dark green) produced by a *Wolbachia*-eliminated mother. Because some of the data contains zeros, 10^−7^ was added to each figure to plot on the log scale. Red dots: ZW individuals (females) of uninfected line. The same letters above the bars indicate no significant difference using generalized linear models with Bonferroni corrections.

Next, we compared the titers of *Osdsx, OsMasc*, and *Osznf-2* within the first-instar *O. scapulalis* larvae, wherein sex-specific death often occurred in *w*Sca-infected and *w*Sca-eliminated broods (18). WZ offspring (female) and ZZ offspring (male) of uninfected females, WZ offspring (female) and ZZ offspring (destined to die) of *w*Sca-infected females, and WZ offspring (destined to die) and ZZ offspring (male) of *w*Sca-eliminated females were compared (Fig. 4). For *Osdsx*, the overall expression levels in the first-instar stage were very low compared to those in the adult stage (cf. Fig. 3). Although not statistically significant (*P* > 0.05), *Osdsx^M^* tended to show slightly higher expression in ZZ than in WZ individuals (Fig. 4A and B), and *Osdsx^F^* tended to show the opposite pattern (Fig. 4A and C). *OsMasc^M^* and *Osznf-2^M^* were expressed in normal males, *w*Sca-infected ZZ (destined to die), cured WZ (destined to die), and cured ZZ, whereas they were strongly suppressed in normal females and *w*Sca-infected females (WZ) (Fig. 4D, E, G, and H). *Osznf-2^F^* expression was upregulated only in normal females and *w*Sca-infected females (WZ) (Fig. 4G and I). *OsMasc^F^* was expressed in all types of individuals (Fig. 4D and F), which is inconsistent with its sex-specific expression at later developmental stages (Fig. 3F; Fig. S5). The fact that both infected ZZ and cured WZ individuals showed upregulation of *OsMasc^M^* and *Osznf-2^M^* expression and downregulation of *Osznf-2^F^* expression suggests that they behave as males rather than females. As reported by Sugimoto and Ishikawa (19), infected and cured WZ have degraded W chromosomes, which are not sufficient for female determination; in infected WZ individuals, *w*Sca is considered to compensate for this insufficiency. However, the male-like expression pattern in infected ZZ individuals suggests that *w*Sca by itself is not sufficient for female determination, suggesting that the combined effects of the degraded W chromosome and *w*Sca are necessary for female determination (or maintenance of femaleness), at least in the first-instar stage.

### Splicing patterns of *Osdsx, OsMasc*, and *Osznf-2* during embryogenesis

**Fig. 5.**
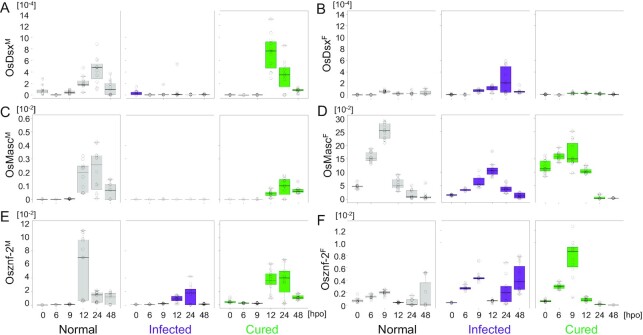
Chronological changes in titers of sex-specific isoforms of *Osdsx, OsMasc*, and *Osznf-2* at 0, 6, 9, 12, 24, and 48 hpo of embryos laid by uninfected females (gray), *Wolbachia*-infected females (purple), and *Wolbachia*-eliminated females (green). For *OsMasc* and *Osznf-2*, different scales were used for the titers of male-specific and female-specific variants (*Y*-axis). Note that both ZW and ZZ embryos are indiscriminately included in every cohort.

Finally, we examined the splicing patterns of *Osdsx, OsMasc*, and *Osznf-2* during embryogenesis, which are most important for sex determination. As expected, *Osdsx^M^* and *Osdsx^F^* showed sex-specific expression during embryogenesis (Fig. 5A and B; Fig. S6), although the titer was much lower than that in later stages. In uninfected embryos (including both ZZ and WZ embryos), *Osdsx^M^* showed a small peak of expression centered around 24 h post oviposition (hpo), which was also the case for the cured broods, whereas all the infected embryos, except for two, exhibited little or no expression (Fig. 5A). By contrast, *Osdsx^F^* showed higher expression in infected embryos than in uninfected and cured embryos during late embryogenesis (Fig. 5B). *OsMasc^M^* and *Osznf-2^M^* showed similar expression patterns, with higher expression during late embryogenesis, i.e., no expression was observed until 12 hpo, after which these isoforms showed a high expression in male embryos of normal broods (Fig. S6) and in most embryos of cured individuals (probably both WZ and ZZ embryos) (Fig. 5C and E). Nevertheless, *OsMasc^F^* and *Osznf-2^F^* isoforms did not show sex-specific expression; their expression levels increased gradually from 0 to 9 hpo in all embryos, before decreasing after 12 hpo, irrespective of sex (Fig. 5D and F). However, after the decrease, *Osznf-2^F^* was highly expressed in some embryos (probably females) at 48 hpo (Fig. 5F), which corresponds to the results of the first-instar stage (Fig. 4F).

In *O. furnacalis*, Fukui et al. (20) concluded that male-specific expression of *OfMasc* was downregulated as a result of *Wolbachia* infection. Our analysis of embryonic RNA-seq data of *O. furnacalis* (DDBJ accession no. DRA003038) revealed the presence of isoforms corresponding to *OsMasc^M^* and *OsMasc^F^*, which were not discriminated in their analysis. We found that the contigs matched to *OsMasc^M^* (the putative *OfMasc^M^*) showed expression peak at 36 hpo in uninfected embryos but almost no expression in *Wolbachia*-infected embryos, whereas the contigs matched to *OsMasc^F^* (the putative *OfMasc^F^*) showed a much higher expression peak at 12 hpo in both infected and uninfected embryos (possibly with slightly lower level in infected embryos) (Fig. S7). The lower level of summed titers of *OfMasc^M^* and *OfMasc^F^* in *Wolbachia*-infected embryos, as represented in Fukui et al. (20), should have led to the irrelevant interpretation that *OfMasc* is male-specific. Expression of *OfMasc^F^* is nonsex-specific and appears to be slightly suppressed in *Wolbachia*-infected embryos at 12 hpo. At 24 hpo or later stages, *Masc* may be sex-specifically expressed. It is probable that during early embryogenesis of *Ostrinia, Wolbachia* suppresses *Masc* (i.e., both *Masc^M^* and *Masc^F^*), but while suppression of nonsex-specific *Masc^F^* is irrelevant to sex determination, suppression of *Masc^M^* leads to female sex determination. The rescue of males through the injection of a complementary RNA of *OfMasc* (20) can be explained by the possible increase in *OfMasc^M^* titers.

### Conclusion and perspective

This study provides compelling evidence that *Wolbachia* infection can manipulate the sex of cultured cells. Notably, this indicates that the *Wolbachia*-mediated host manipulation that we observed was cell-autonomous. Through comparative transcriptomics using *Wolbachia*-infected and uninfected cells, we successfully isolated two genes (*OsMasc* and *Osznf-2*) that changed their splicing patterns after *Wolbachia* infection. Expression profiles during the postembryonic development of *O. scapulalis* revealed that the differences in splicing patterns of these genes were attributed to sex differences (sex-specific splicing). It follows that *Wolbachia* alters the splicing of *OsMasc* and *Osznf-2* in males from male- to female-specific. This feminizing effect is most likely the foundation of the male-killing mechanism in *Ostrinia* (18–20), *Homona* (21), and other lepidopteran insects (35). Our study using male cell lines may facilitate new biochemical and molecular investigations into *Wolbachia*-induced sex manipulations in various insect species. Expression patterns during embryogenesis suggest that male-specific splice variants of *OsMasc* and *Osznf-2* play an important role in initial sex determination, whereas female-specific variants of these genes do not. Moreover, in the first-instar stage of *Wolbachia*-infected matriline, the combined action of the W chromosome and *Wolbachia* appeared to be necessary for proper female-specific splicing of *OsMasc* and *Osznf-2*, suggesting reciprocal interdependence of the degraded W chromosome and *Wolbachia*. The persistence of the W chromosome in the *Wolbachia*-infected *O. scapulalis* line is contrary to the loss of the W chromosome in the *Wolbachia*-associated all-female matriline of *Eurema* butterflies (36). Incomplete transfer rather than complete transfer of the W chromosome roles to *Wolbachia* may have hindered the loss of the W chromosome in *O. scapulalis*, and this may have resulted in the stable coexistence of the W chromosome and the feminizing *Wolbachia*.

When a male-killer increases in frequency among the host population [although this is not the case for *Ostrinia* moths, wherein male-killers are maintained in low frequencies (37, 38)], suppressors of male-killing are expected to evolve in the host (35). Fixation of suppressors has been observed in real time in butterflies and lacewings (39, 40), whereas in several other systems, observations have been made after suppressor fixation (30,41–43). Therefore, absence of male-killing in native hosts does not necessarily mean that the *Wolbachia* does not have male-killing ability. Our study highlights the possibility of identifying the inherent ability for male-killing in each of the *Wolbachia* strains harbored by various arthropod hosts by examining the gene expression changes in insect cells. The findings would help us determine, in terms of insect genome dynamics, the frequency at which male-killing suppressors evolved in the face of male-killing *Wolbachia*.

We also point out the fact that, without any a priori functional or sequence information, sex-determining genes were clearly isolated from tens of thousands expressed genes. Hence, we believe that our approach using cell lines would be effective in identifying novel sex-determining genes of nonmodel insects, which had been challenging by using organismal samples.

## Material and Methods

### Rearing of *O. Scapulalis*

Larvae were fed with commercial diet Silkmate-2 M (Nihon Nosan, Yokohama, Japan). Insects were reared at 23°C under the 16 h/8 h of light/dark photoperiod. Elimination of *Wolbachia* from infected *O. scapulalis* was achieved by rearing the entire larval stage on Silkmate-2 M supplemented with 0.06% tetracycline hydrochloride (CDX-T0096, Funakoshi, Japan) (37, 38).

### Primary cell culture of *O. Scapulalis*

We attempted to establish cell lines from an uninfected (*Wolbachia*-free) strain of *O. scapulalis* using the culture media used for primary cell culture are either MGM-464 (44) containing 20% heat-inactivated fetal bovine serum (Corning Inc., USA) or the MX20 (US Pat. No. 7074612) (Fig. S1). Each medium was supplemented with 0.1% polyvinylpyrrolidone K-90 (FUJIFILM Wako Pure Chemical Corp. 168–17042, Japan), 1 mg/ml reduced glutathione (FUJIFILM Wako Pure Chemical Corp. 073–02013), and 10% antibiotics–antimycotics (Gibco 15240–096; Themo Fisher Scientific, USA). Final-instar larvae and pupae of *O. scapulalis* were sterilized in 70% ethanol for 10 min and air-dried on the sterilized paper towel in the laminar flow cabinet. Larvae and pupae were then anchored on sterilized silicon plates, and fat bodies and testes were dissected out and transferred to new medium drops individually. Sex was determined by the presence or absence of testes. After rinsing twice in a fresh medium, the fat bodies and testes were cut into several pieces using a thin blade and placed with a 500-µl medium in a 12.5-cm^2^ culture flask (Falcon^®^ 353018; Corning Inc.), which was coated with poly-L-lysine (PLL) (Sigma-Aldrich P4707; Merck KGaA, Germany) following Watanabe et al. (45).

In the primary culture of larval fat bodies, many round cells started to migrate after 1 week of incubation and continued to proliferate and reached confluence in a flask in the following week. In the primary culture of larval and pupal testes; however, only a few round cells were released after 1 week and finally reached confluence after 5 months. The culture was maintained at 25°C. Approximately, one-third of the medium was replaced with a fresh medium every 10 to 14 d. One month after initiation, the medium was replaced with a polyvinylpyrrolidone-free, glutathione-free, and antibiotic–antimycotic-free medium. After first subculturing, PLL-noncoated flasks were used. The medium was replaced with MGM-450 (46) supplemented with 10% fetal bovine serum at approximately 600 days after initiation.

### Growth curves of the established cell lines

To examine the growth curves, 1.2 × 10^6^ cells prepared in a centrifuge tube was centrifugated at 300 × *g* for 3 min, and the supernatant was removed. After resuspended with 12 ml of a fresh medium, a 500 µl of the cell suspension was aliquoted into each well of the 24-well cell culture plate (SUMILON, Cat. No. MS-80240; SUMITOMO BAKELITE Co., Ltd) (final concentration: 1 × 10^5^ cells per ml). Cell counts were made 1, 2, 4, and 7 d after subculturing, using 10 µl of the cell suspension taken from each of the selected three wells by using a cell counter plate (Cat. No. 177–112C; WATSON, Japan). To calculate population doubling time (PDT), we used the following expression: PDT = (t−t_0_) log2/(logN—logN_0_) (47).

### Transinfection of *Wolbachia* into cultured cells

Fat bodies, septically isolated from *Wolbachia* (*w*Sca)-infected *O. scapulalis*, were placed in NARO-Bm-M2, a *B. mori* cell line established from male embryos. After confirming by diagnostic PCR that *w*Sca was stably maintained, the *w*Sca-positive NARO-Bm-M2 was used as a donor of *Wolbachia* transinfection into *O. scapulalis* cell lines. Cells harvested from *w*Sca-infected NARO-Bm-M2 were passed through a 5.0-µm filter (GVS, Cat. No. FJ25ASCCA050PL01), and six drops (ca. 300 µl) of the flowthrough were added to each of the recipient cell lines (i.e., M1, M4, M7, M8, and F6).

### Elimination of *Wolbachia* from cell lines

For elimination of *Wolbachia* from *O. scapulalis* cell lines, 1 ppm of tetracycline hydrochloride was added to the cell culture medium MGM-450.

### RT-PCR

Total RNA, extracted using TRIzol RNA Isolation Reagents (Thermo Fisher Scientific, USA) followed by precipitation using 2-propanol and rinsing with 75% ethanol, was subjected to reverse transcription by PrimeScript II 1st strand cDNA Synthesis Kit (Takara Bio, Japan) using random 6mers. The PCR reaction was performed using KOD FX Neo (Toyobo, Japan).

### RNA sequencing

The uninfected M1, M7, and M10 and the *w*Sca-transinfected M1, M7, and M10 (six cell lines in total) were used for RNA-seq analysis. The cell lines were maintained at 25°C. One month after transinfection, each cell line was subjected to total RNA extraction using RNeasy Plus Mini Kit (Qiagen, Germany) according to the manufacturer instruction. RNA concentration and purity were checked with a NanoDrop Lite spectrophotometer (Thermo Fisher Scientific, USA). Total RNAs were used for preparation of a library suitable for Illumina Hiseq pair-end (2× 100 bp) sequencing (TruSeq RNA Sample Prep Kit v2, Illumina, USA). RNA sequencing was performed by Macrogen (South Korea) using Illumina Hiseq 2500 System. The raw sequence data were deposited as a Sequence Read Archive at National Center for Biotechnology Information (accession number DRA014263).

### Bioinformatics analysis

Raw FASTQ sequence data was imported to usegalaxy.org web server (https://usegalaxy.org). Trim Galore! (Galaxy Version 0.6.3) with Cutadapt was applied to remove adapter sequences and reads with low quality (Qscore < 20) and reads containing *N* < 90. The quality of the trimmed reads was assessed using FastQC. Trimmed paired reads in each library were aligned to the *O. furnacalis* assembly genome using RNA STAR aligner in the 2-pass mapping mode with the following parameters—Multimap Score Range: 1, Multimap N Max: 2, Mismatch N Max: 2, Maximum ratio of mismatches to mapped length: 0.1, and other parameters were set to default. By using the software Stringtie, genome guided assembly was made for each RNA-seq sample by using genome data of *O. furnacalis*. The outputs were combined as a single file using Stringtie merge and was output as gff3 file containing new transcript data suitable for the analyses. Htseq-count data used to estimate the transcript titers were subjected to Deseq2 for comparative analyses and heatmap2 for visualization. All the analyses were performed with the default setting unless otherwise noted.

### Quantitative RT-PCR

Harvested cell or insect materials were subjected to RNA extraction using Trizol (Thermo Fisher Scientific). Reverse transcription was made using PrimeScript II 1st strand cDNA Synthesis Kit (Takara), RNA 200–500 ng, 1 µl dNTP Mixture (10 mM each), 1 µl random hexamers (50 μM), filled with RNase-free water up to 10 µl total volume. The qPCR reaction was made with 5 µl KOD SYBR (Toyobo), 0.4 µL forward primer [10 pmol/µl], 0.4 µl reverse primer [10 pmol/µl], and 2.2 µl water. The qPCR reactions were made in LightCycler 96 System (Roche) with the temperature profile of 180 s at 95°C, 40 cycles of 8 s at 98°C, 10 s at 60°C, and 10 s at 68°C, followed by heating to 90°C for a melt curve analysis. Primers used in this study are listed in Table S1.

### Cytogenetic sexing

While pupae and adults were sexed based on morphological characters, larvae of all stages were sexed by the cytogenetic method following Kageyama and Traut (18). Each larva placed on a glass slide was teared apart using fine forceps under a dissecting microscope. Several filamentous tissues, either silk glands or Malpighian tubules, were picked up and placed on a new glass slide, and the remains were soaked into 100-μl TRIzol reagent aliquoted in 1.5-ml microtube, which was then flash-frozen in liquid nitrogen and stored at −80°C prior to RNA extraction. A few drops of freshly prepared methanol: acetic acid (3:1) were placed on the filamentous tissues on the glass slide. After 1 min, the preparation was stained and mounted in lactic acetic orcein. After 3 min, the preparation was observed under the light microscope DM500 (Leica Microsystems). Sex chromosome constitution (i.e., WZ or ZZ) was inferred by the presence or absence of condensed chromatin body (W chromosome) in highly polyploid interphase nuclei.

### Statistical analysis

The relative transcript titers estimated by quantitative RT-PCR were subjected to statistical analyses using Software R ver. 4.0.4 (48). Multiple comparisons were performed with Bonferroni corrections. As some of the data sets did not exhibit a normal distribution and/or homogeneous variance, we adopted the generalized linear model (49) for Gaussian, inverse Gaussian, gamma, or negative binomial distributions, which was selected according to the Akaike information criterion.

## Supplementary Material

pgac293_Supplemental_Files

## Data Availability

All data are included in the manuscript and/or Supplementary Material.
