# Supplementary material for: Cell-based analysis reveals that sex-determining gene signals in Ostrinia are pivotally changed by male-killing Wolbachia
Source: PNAS Nexus. 2022 Dec 13;2(1):pgac293. doi: 10.1093/pnasnexus/pgac293 (PMC9837667; doi:10.1093/pnasnexus/pgac293)
Supplement: pgac293_Supplemental_Files [file pgac293_supplemental_files.zip › PNASNEXUS-PNASNEXUS-2022-00875-s01.pdf]

Supplementary figures for

Cell-based analysis reveals that sex-determining gene signals in *Ostrinia* are pivotally changed by male-killing *Wolbachia*

Authors:
Benjamin Herran[a,1,†], Takafumi N. Sugimoto[a,1,*], Kazuyo Watanabe[a], Shigeo Imanishi[a], Tsutomu Tsuchida[b], Takashi Matsuo[c], Yukio Ishikawa[d], and Daisuke Kageyama[a,*]

Affiliations:
[a]Institute of Agrobiological Sciences, National Agriculture and Food Research Organization, 1-2, Owashi, Tsukuba, Ibaraki 305-0851, Japan
[b]Toyama University, 3190, Gofuku, Toyama 930-8555, Japan
[c]Graduate School of Agricultural and Life Sciences, University of Tokyo, 1-1-1, Yayoi, Bunkyo-ku, Tokyo 113-8657, Japan
[d]Faculty of Agriculture, Setsunan University, 17-8, Ikedanakamachi, Neyagawa, Osaka 572-8508, Japan

†Present address: Faculty of Fundamental and Applied Sciences of Nantes University, 2 chemin de la Houssinière, 44322 Nantes, France.
[1]These authors contributed equally to this work.

[*]To whom correspondence should be addressed. Email: sugimotot032@affrc.go.jp or kagymad@affrc.go.jp

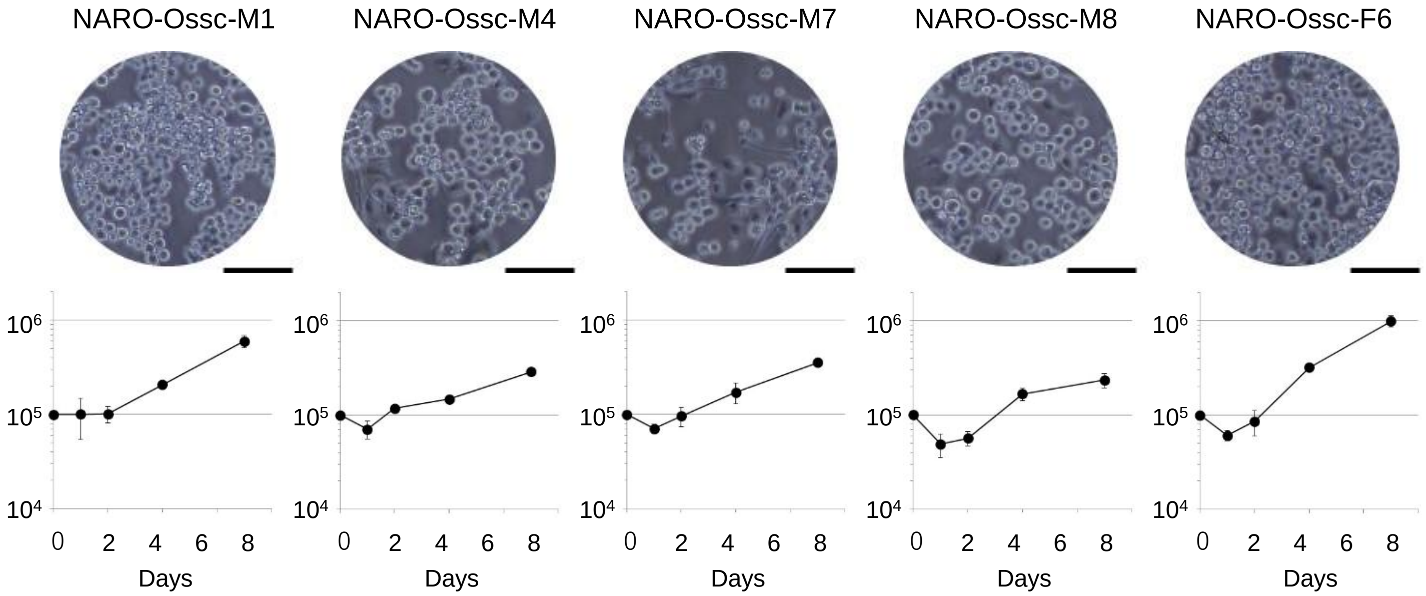

Cell lines	Origin			Initiation medium	50 passages (days)	PDT (days)
	Tissue	Stage	Sex			
NARO-Ossc-M1	Fat bodies	Last-instar larva	Male	MGM-464, 20%FBS	666	2
NARO-Ossc-M4	Testes	Pupa	Male	MX20	840	3.8
NARO-Ossc-M7	Testes	Last-instar larva	Male	MGM-464, 20%FBS	771	2.7
NARO-Ossc-M8	Fat bodies	Last-instar larva	Male	MGM-464, 20%FBS	722	2.4
NARO-Ossc-F6	Fat bodies	Last-instar larva	Female	MGM-464, 20%FBS	596	1.7

Figure S1. Five cell lines of *O. scapulalis* and the growth curves. Bar: 100 μm. Numbers of cells per milliliter were counted for three times. Means ± standard deviations are shown. Origin of the cell lines, initiation medium, days taken for the first 50 passages, and the population doubling time (PDT) are given in the table.

Gene_id	Mean counts	log2(FC)	StdErr	Wald_ Stats	P-value	Adjusted P-value
MSTRG.20807	116.05	4.03	0.44	9.13	7.06×10^{-20}	2.47×10^{-15}
MSTRG.20794	162.42	-3.06	0.45	-6.73	1.71×10^{-11}	2.99×10^{-7}
MSTRG.20809	73.89	3.23	0.49	6.59	4.26×10^{-11}	4.97×10^{-7}
MSTRG.20791	195.35	-2.46	0.39	-6.38	1.80×10^{-10}	1.57×10^{-6}
MSTRG.30473	20.87	-2.34	0.47	-4.98	6.33×10^{-7}	4.43×10^{-3}

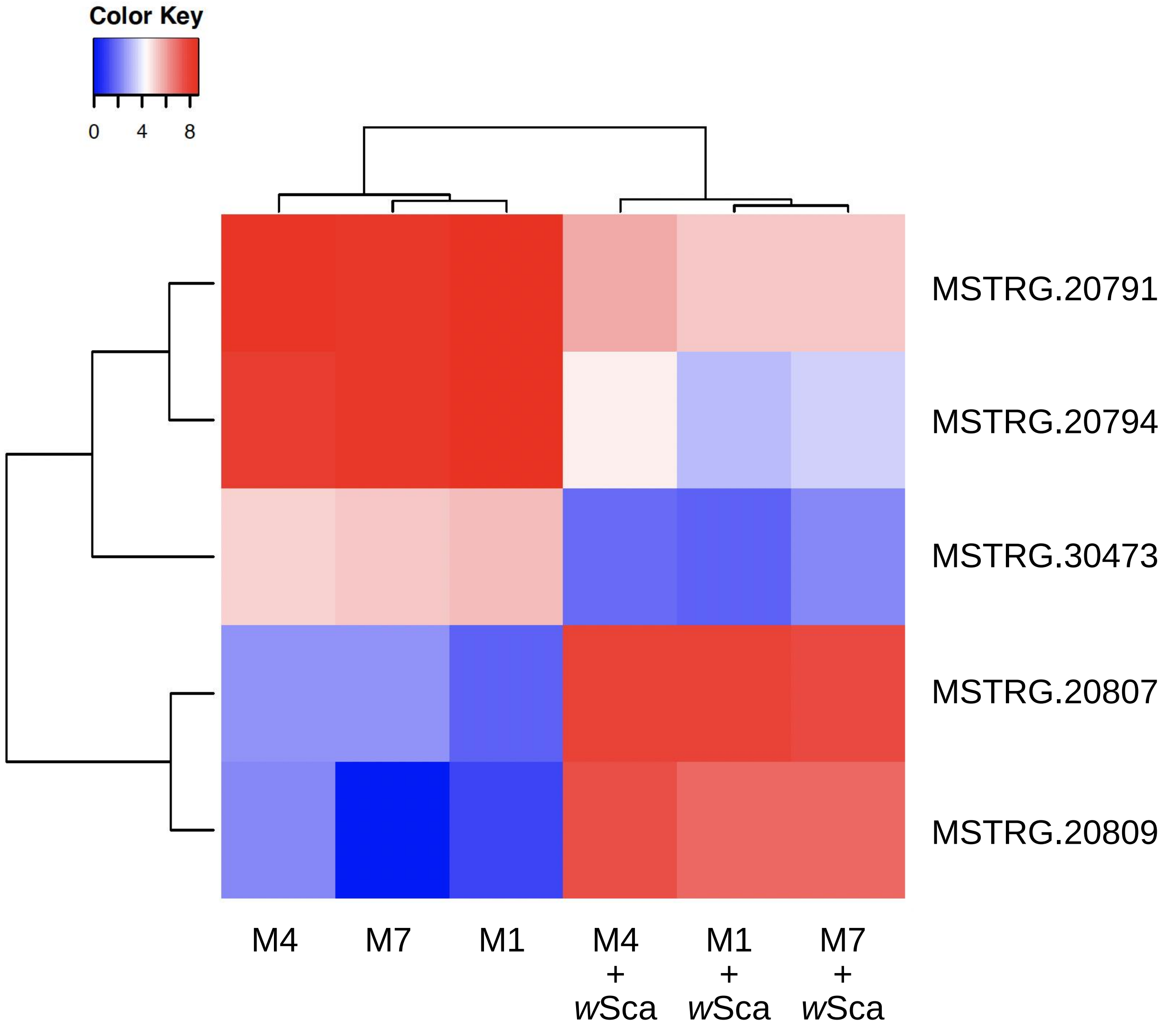

Figure S2. Differentially expressed contigs between the control and *w*Sca transifected cells detected by Deseq2 analysis. Cut-off value of the adjusted P-value was set as 0.1. The Heatmap is about the five contigs that showed significant upregulation/downregulation upon *w*Sca infection.

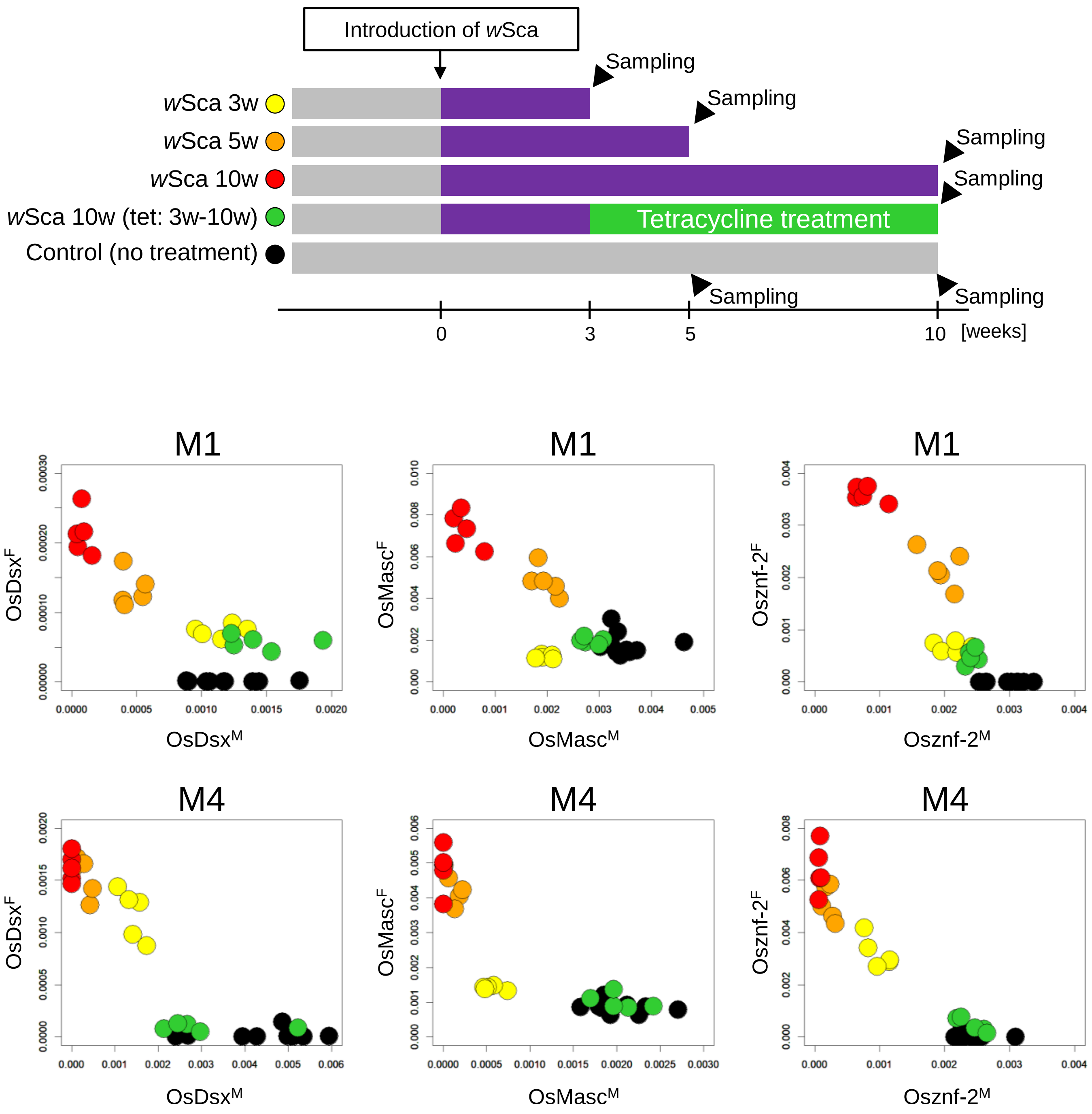

Figure S3. Relative titers of the sex-specific isoforms of *Osdsx*, *OsMasc*, and *Osznf-2* in male-derived *O. scapulalis* cell line M1 and M4 (cf. Fig. 2 for M4). Purple bars represent the duration after *w*Sca transinfection. Green bar represents the duration of tetracycline treatment. See text for details.

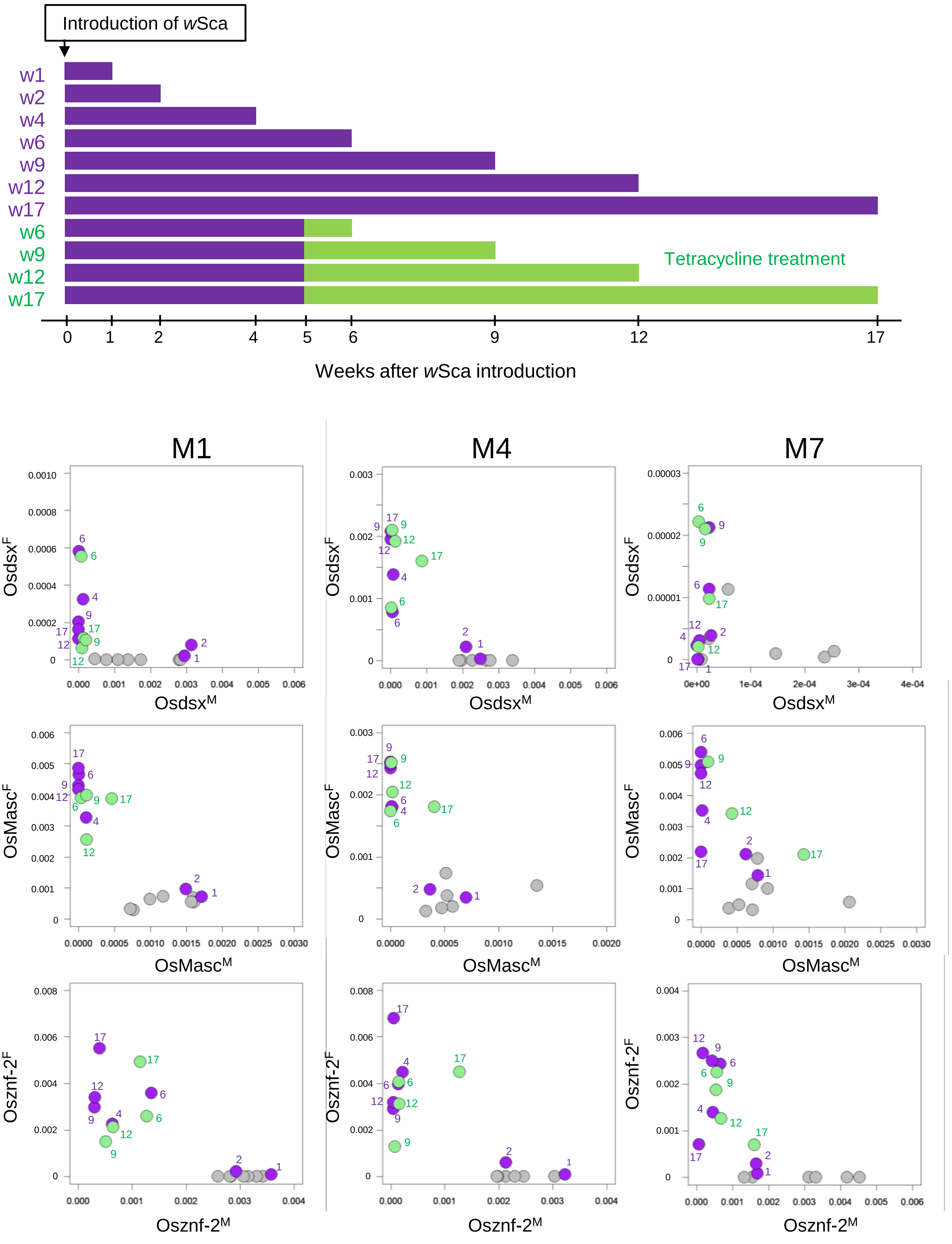

Figure S4. Relative titers of sex-specific isoforms to *Osdsx*, *OsMasc*, and *Osznf-2* in male-derived *O. scapulalis* cell lines M1, M4, and M7. Purple bars represent the duration after *w*Sca transinfection. Green bars represent the duration of tetracycline treatment.

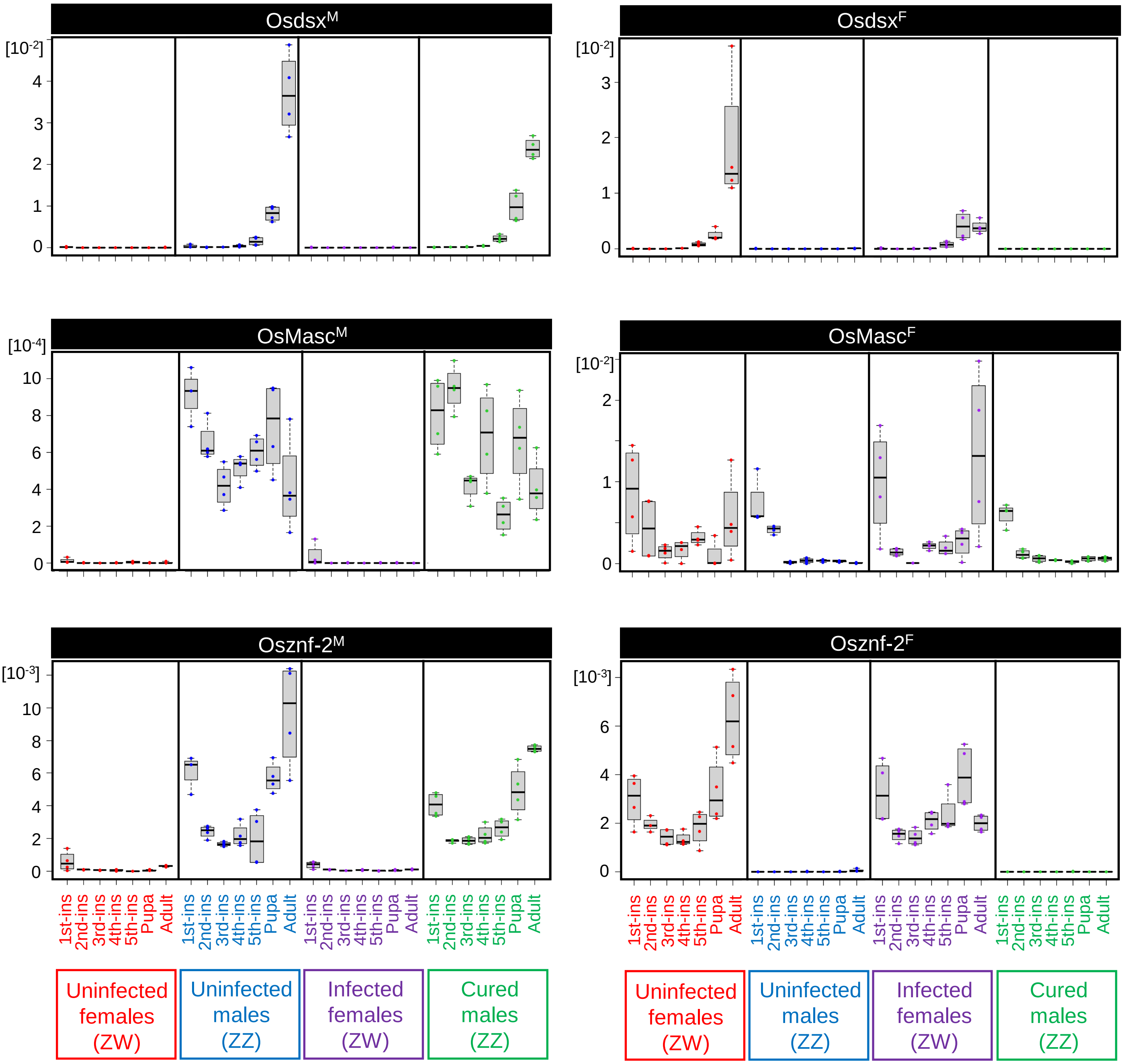

Figure S5. Relative titers of the sex-specific isoforms of *Osdsx*, *OsMasc*, and *Osznf-2* during development of *O. scapulalis*.

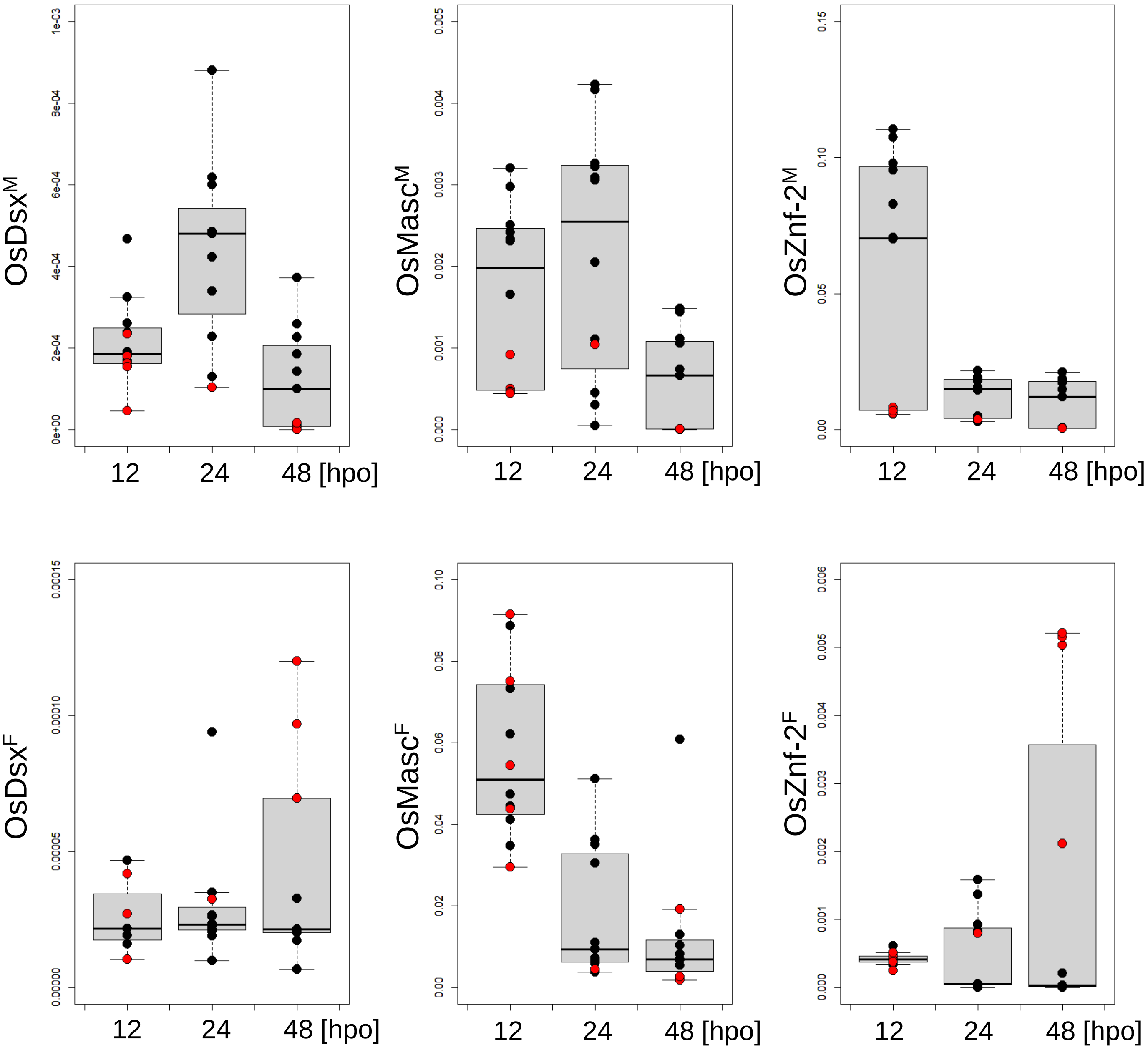

Figure S6. Relative titers of the sex-specific isoforms of *Osdsx*, *OsMasc*, and *Osznf-2* during embryogenesis (12, 24, and 48 hpo) of the uninfected strain of *O. scapulalis*. A red dot shows an individual embryo positive for the female-specific genomic PCR, which works only for the uninfected strain, and this, considered as ZW female. A black dot shows an individual embryo negative for the female-specific genomic PCR, and thus considered as ZZ male. It is possible that some of the DNA samples were low in quality or quantity, which hampered the PCR amplification and incorrectly regarded as male (black dots).

Putative *OfMasc*^{M}

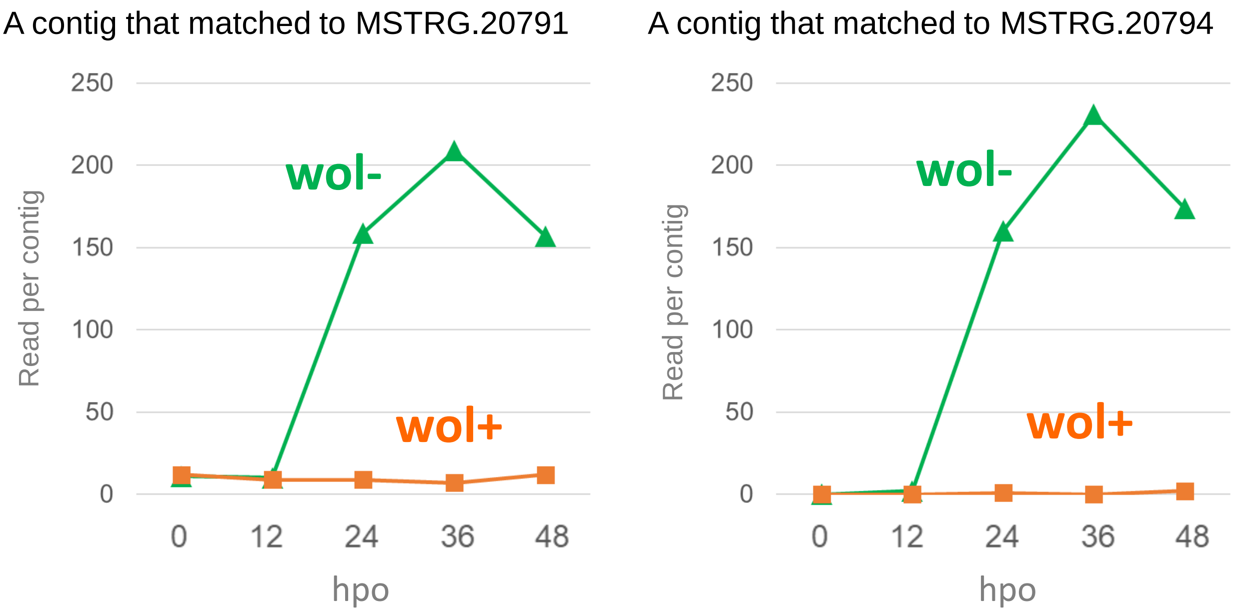

Putative *OfMasc*^{F}

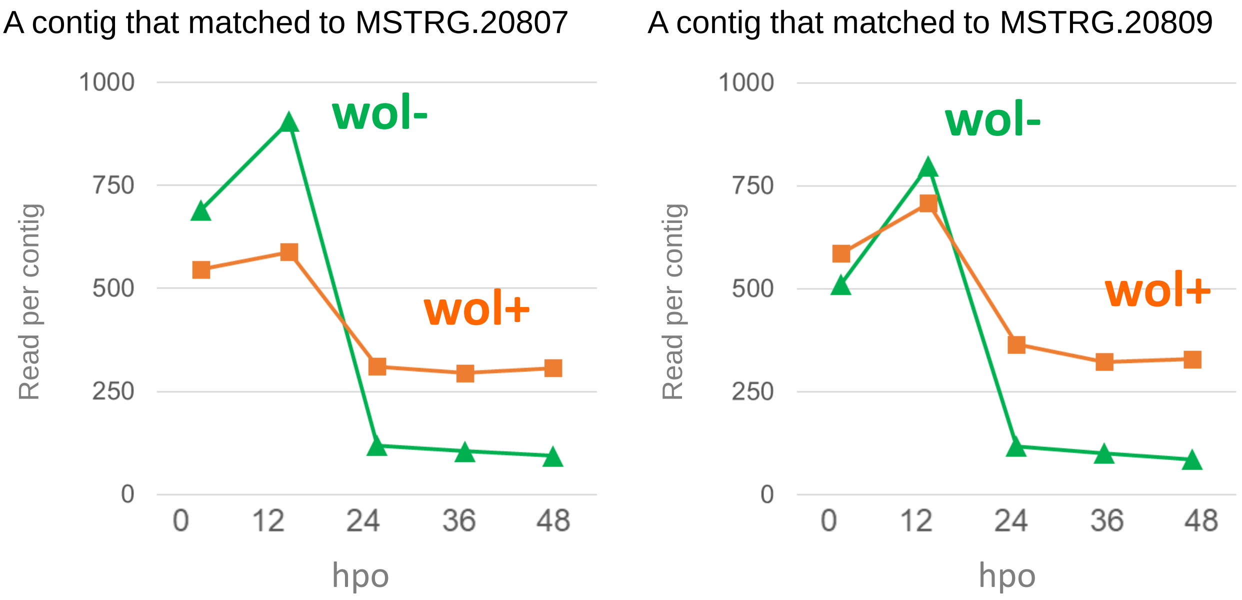

Figure S7. Read counts of the four RNA-seq contigs of *Wolbachia*-infected and uninfected *Ostrinia furnacalis* embryos (0, 12, 24, 36, and 48 hpo) that match to the sex-specific isoforms (MSTRG.20791, MSTRG.20794, MSTRG.20807, and MSTRG.20809) of *OsMasc*. Raw data of RNA-seq [DRA003038] published by Fukui et al. (2015) was analyzed.

Table S1. Primers used in this study.

Target	Primers	Primer sequences (5'-3')	Primer size (bp)	Amplicon size (bp)	Remarks
Dsx^M	OsDsxM-qF1	GAGGAAGATTGATGAAGCCCAC	22	264	Overlapping introns + female-specific exon
	OsDsxM-qR1	GACGGAGGCTCTGATGACTC	20		Common
Dsx^F	OsDsxF-qF3	CGAGGAAGATTGATGAAGGGAAG	23	116	Overlapping intron
	OsDsxF-qR2	GCTTTGCAGCATTTTCTGGC	20		Female-specific
$Masc^M$	OsMascM-qF1	GCCAAATGGACATTACAACCAGTAC	25	113	Common
	OsMascM-qR1	CACGACTCGTGTCGACCAAAC	21		Male-specific + far from forward in genome
$Masc^F$	OsMascF-qF1b	TTATATCAGGGGTGGCCTACT	21	187	Female-specific
	OsMascF-qR3	TCAACTATATAATTCAGGTGTGGC	24		Female-specific + overlapping intron
$Znf2^M$	OsZnf2M-qF1	CCACCGAATCAAGCAATTGC	20	145	Male-specific + overlapping intron
	OsZnf2M-qR1	TTTTCGTTCGCTTCGGATATTTG	23		Male-specific
$Znf2^F$	OsZnf2F-qF3	AGTGTTCTGTGGTAATTAATTCGC	24	141	Common (5'-UTR)
	OsZnf2F-qR2	GGGCGCGGCCGTTGATTC	18		Overlapping introns + male-specific exon
RPS3	OsRP3-qF	TGCTATGGTGTGCTGAGGTTC	21	146	Internal control
	OsRP3-qR	TAGTCGTTGCATGGGTCTCC	20		
EF1α	Os-EF1a-F	GACTCCGGCAAGTCCACCAC	20	94	Internal control
	Os-EF1a-R	CCTGGGCCTCCTTCTCGAAT	20		
